# Chitosan-Graft-Poly(N-Isopropylacrylamide)/PVA Cryogels as Carriers for Mucosal Delivery of Voriconazole

**DOI:** 10.3390/polym11091432

**Published:** 2019-08-31

**Authors:** Catalina Natalia Cheaburu-Yilmaz, Onur Yilmaz, Fadime Aydin Kose, Nela Bibire

**Affiliations:** 1 Department of Physical Chemistry of Polymer “Petru Poni” Institute of Macromolecular Chemistry, 700487 Iaşi, Romania; 2 Ege University, Faculty of Engineering, Leather Engineering Department, 35100 Bornova-Izmir, Turkey; onur.yilmaz@ege.edu.tr; 3 Ege University, Faculty of Pharmacy, Department of Biochemistry, 35100 Bornova-Izmir, Turkey; fadime.aydin@ege.edu.tr; 4 Department of Analytical Chemistry, Faculty of Pharmacy, Grigore T. Popa University of Medicine and Pharmacy, 700115 Iaşi, Romania

**Keywords:** chitosan, graft copolymer, CS-*g*-PNIPAAm, pH and temperature responsive, cryogel, freeze–thaw, gel texture, formulation, Voriconazole

## Abstract

The objective of this study was to prepare and characterize physically crosslinked gel formulations of chitosan (CS)-graft-poly(N-isopropyl acrylamide) (PNIPAAm) and polyvinyl alcohol (PVA) for smart delivery of an antifungal drug, Voriconazole, for mucosal applications. For this purpose, cryogels of CS-*g*-PNIPAAm/PVA and CS/PVA were tested by means of texture profile analysis and rheology to determine optimal matrix properties for topical application. The ratio of 75/25 *v*/*v* % CS-*g*-PNIPAAm/PVA was selected to be used for formulation since it gave low compressibility and hardness (1.2 and 0.6 N) as well as high adhesion properties and non-Newtonian flow behavior. The cryogels and formulations were further characterized by means of FTIR spectroscopy, swelling behavior, texture analysis, scanning electron microscopy (SEM), thermal (differential scanning calorimetry (DSC) and TGA), and rheological behavior. The drug loading capacity and in vitro release profile of the drug, storage stability, and cytotoxicity tests were also performed for the gel formulation. The FTIR, DSC, and TGA results verified the successful formation of cryogels. Swelling studies revealed a pH-dependent swelling ability with a maximum swelling degree of 1200% in acid and 990% in phosphate buffer (pH = 7.4). Thermal studies showed that CS-*g*-PNIPAAm/PVA 75/25 had higher thermal stability proving the structural complexity of the polymer. The loading capacity of Voriconazole was found to be 70% (*w*/*w*). The in vitro release profiles of Voriconazole showed Fickian release behavior for CS-*g*-PNIPAAm/PVA 75/25 gel with an approximate delivery of 38% within 8 h, slower than matrices containing unmodified chitosan. The storage stability test exhibited that the gel formulation was still stable even after aging for two months. Moreover, the cell culture assays revealed a non-toxic character of the polymeric matrix. Overall results showed that the CS-*g*-PNIPAAm/PVA 75/25 hydrogel has the potential to be used as a smart polymeric vehicle for topical applications.

## 1. Introduction

Polysaccharides represent a distinctive class of materials preferably used in the preparation of pharmaceutical formulations as drug carriers. Chitosan, alginate, hyaluronic acid, etc., are the most favored polysaccharides used in pharmaceutical science for drug delivery systems due to their special properties such as biocompatibility, bioadhesivity, and biodegradability. 

Chitosan, as a natural based polymer, has attracted great interest in the last decades. Its main drawback, poor solubility in water and organic solvents, made it a subject of many challenging studies. Most of the reports dealt with the chemical modification of chitosan in order to obtain chitosan derivatives with enhanced solubility in various solvents and improved characteristics [1,2,3,4].

Chitosan has a highly regular structure consisting of *β*-(1→4) linked D-glucosamine repeating units with three reactive groups, giving many possibilities of functionalization. Among the various methods, graft modification is considered as a convenient and promising approach to expand the applicability and functionality of chitosan [2,3,4,5,6,7,8]. However, the conventional grafting techniques on chitosan do not give control on the side polymer chain lengths and composition, the number of grafting sites, and grafting percentage. Despite the great interest for conventional graft modification, controlled/living free radical polymerization techniques seem more attractive nowadays because they offer the possibility of synthesizing polymers with well-controlled structures under relatively simple experimental conditions [9,10]. 

Cheaburu-Yilmaz et al. [11] reported the mechanisms for reversible addition–fragmentation chain transfer (RAFT) polymerization/grafting of different monomers onto the chitosan backbone. Various attempts at chitosan chemical modification were also presented within the same review study. The most successful way to obtain a modified chitosan was shown to be via the grafting of a pH- or temperature-responsive polymer on primary –OH groups at the C2 position. The resulting chitosan derivative with free NH_2_ groups at C6 and responsive side chains at C2 can be a good candidate for advanced drug vehicles. In another study by Cheaburu-Yilmaz [12] chitosan grafted PNIPAAm (CS-*g*-PNIPAAm) copolymers were prepared via reversible addition–fragmentation chain transfer (RAFT) polymerization subsequent to the attachment of 4-cyano-4-(phenylcarbonothioylthio) pentanoic acid on chitosan –OH groups and to use chitosan as macro chain transfer agent. The grafting efficiency was quantified as being 40% and the final copolymer was water soluble and possessed pH and temperature sensitivity. However, the water solubility and responsiveness of the copolymer might not guarantee to fulfill all the requirements of being an ideal drug carrier for mucosal treatment. The copolymer solutions needed to have enhanced viscosity or a certain gel character in order to be attached or to adhere on the surface of mucosa. An alternative strategy was blending the synthesized copolymer with a hydrogen-bonding capable polymer to make physical crosslinks with the NH_2_ groups of CS-*g*-PNIPAAm. The best choice was polyvinyl alcohol (PVA), a linear polymer, which can easily interact with the functional groups of graft copolymer. More particularly, when PVA solutions are exposed to alternative cycles of freezing at −20 °C and then thawing at room temperature, network-like, inter/intra molecular entanglements are formed leading to physical crosslinking [13,14,15,16,17,18]. Crosslinking of chitosan derivatives to obtain gels is widely used, especially with external crosslinkers (i.e., aldehydes), which usually exhibit a harmful and/or toxic character in nature [19]. On the other hand, crosslinking with the freeze–thaw process is simple, easy to perform, and it does not need addition of any crosslinking agent to prepare gels [18,20]. 

The antifungal Voriconazole (Vor), a second-generation triazole with a large spectrum of action is one of the most recommended systemic antifungal agents as the first line therapy against several types of systemic mycoses, including *Candida albicans* [21,22]. Due to the side effects of Voriconazole through oral and/or intravenous administration such as photopsia,  abdominal  pain,  and  visual  hallucinations, a need exists for a topical delivery system for Voriconazole to overcome limitations and which will  be  particularly  useful  against  candidiasis  in  wounds  and skin tissue [23,24]. 

The present study deals with the preparation of CS-*g*-PNIPAAm/PVA cryogel via a physical crosslinking procedure as an ecological and efficient approach. The optimized hydrogel was used as a matrix for the smart delivery of Voriconazole, for mucosal application. The cryogels and optimized formulation were characterized in detail by means of chemical structure, thermal and rheological behavior, pH and temperature sensibility, cytotoxicity and stability, etc., and the results were discussed. 

## 2. Materials and Methods 

### 2.1. Materials 

In the experiments, a previously synthesized [12] chitosan-graft–poly(N-isopropylacrylamide) copolymer (CS-*g*-PNIPAAm) was used in the preparation of cryogels. Polyvinyl alcohol (PVA) 99% hydrolyzed with *M*_w_ of 89,000–98,000 Da was purchased from Sigma-Aldrich, St. Louis, MO, USA. Phosphate buffered saline pH = 7.4 (PBS) was purchased from Sigma-Aldrich, St. Louis, MO, USA, as tablets. As the active substance, Voriconazole, a Sigma product, Steinheim, Germany was used. 

### 2.2. Preparation of Freeze–Thaw Hydrogels CS-g-PNIPAAm/PVA

Freeze–thaw hydrogels were prepared by mixing 1 wt.% water solution of CS-*g*-PNIPAAm and 5 wt.% PVA solution in a volume ratio of 75/25 *v*/*v* %. Solutions were frozen at −20 °C for 20 h and thawed at 25 °C for 4 h with three cycles. Preliminary texture profile analysis (TPA) and rheology tests were done to select the best composition for a proper topical gel application demanding low hardness and high adhesion properties. Additionally, for this purpose, freeze–thaw hydrogels from unmodified chitosan with PVA were prepared as reference with the same compositions. Table 1 summarizes the list and the codes of the prepared gels. 

### 2.3. Preparation and Optimization of Pharmaceutical Formulation

Semisolid pharmaceutical dosage forms were prepared by mixing the active substance together with the polymeric carrier and water forming a gel. Gel formulations were prepared as such to contain 5 wt.% polymer content; this concentration represented an optimal concentration which was pre-determined based on the texture profile properties of the gels with different concentrations (not shown herein). 

As an outcome of the preliminary tests, CS-*g*-PNIPAAm/PVA 75/25 was selected as the polymeric vehicle for Voriconazole for topical application. 

Loading of Voriconazole was done by mixing the graft copolymer solution with Voriconazole 1 wt.% in an aqueous solution of acetic acid 0.5 wt.% with 1 mL of methanol for a better dissolution of the drug. A solution of PVA, 5 wt.%, was added and freeze–thawing cycles were performed as mentioned above. The final gels were lyophilized. The pH of the gel formulation was determined as 5. 

### 2.4. Characterization Methods

#### 2.4.1. FTIR Spectroscopy

The chemical characterization of the obtained intermediary and final products was performed by using a Perkin Elmer Spectrum-100 instrument, Shelton, CT, USA, through reflection on a diamond crystal with an angle of 45°, resolution of 4 cm^−1^ and scanned between 400 and 4000 cm^−1^.

#### 2.4.2. Swelling Behavior of the Polymeric Hydrogels

The swelling capacity of the freeze-dried hydrogels was investigated by direct immersion of formulations in acidic medium, water, and phosphate buffer saline (PBS, pH = 7.4) to simulate medium conditions for in vitro experiments. The samples were periodically removed from the solution, gently wiped with a soft tissue to remove surface solution, weighed, and then carefully placed back into the vessel as quickly as possible.

The swelling degree (Q) was calculated according to Equation (1):

(1) $$Q\;\left(\%\right)=\frac{\left(W_{t}-W_{d}\right)}{W_{d}}\times 100,$$

where *W_t_* is the weight of the swollen samples at time t and *W_d_* is the weight of the dry sample.

#### 2.4.3. Rheological Measurements

The rheological investigations were performed by using an Anton Paar MCR301 Rheometer, Berlin, Germany equipped with a cone-plate geometry measuring system with a cone angle of 1° and a diameter of 50 mm at different shear rates and angular frequencies. The samples were placed onto the plate for 5 min to eliminate residual shear history, and then experiments were carried out immediately. The measuring device was equipped with a temperature unit that gave good temperature control (±0.05 °C). The rheological tests were done as follows:

(i) Flow behavior (Newtonian or non-Newtonian): The flow behavior was tested by rotational controlled shear rate condition (CSR), where the viscosity (η) of the samples were measured as a function of increasing shear rate (*γ* = 0.1–1000 s^−1^).

(ii) Viscoelastic behavior: Dynamic modulus, storage modulus (G’), and loss modulus (G’’) of the hydrogels were measured as a function of angular frequency (ω = 0.1–1000 s^−1^) using oscillatory tests. To perform the frequency sweep tests, the linear viscoelastic range of the samples (LVE) was obtained from amplitude sweep tests (with a strain amplitude between 0.01% and 500%) using a constant angular frequency ω = 10 s^−1^.

(iii) Temperature-dependent behavior of hydrogels: This type of oscillatory test was performed maintaining both frequency and amplitude constant, the only variable parameter being temperature. The temperature was varied within 25–55 °C and the storage modulus values were plotted against temperature. The sol–gel transition temperature *T_g_* of the gels was measured. 

(iv) Stability tests: The rheological properties of hydrogels before and after the aging process were measured. The dynamic moduli and sol–gel temperature of the samples were compared.

#### 2.4.4. Thermogravimetric Analysis (TGA)

Thermogravimetry was performed by using a Perkin Elmer TGA 4000, Waltham, MA, USA, device to evaluate the thermal degradation process of the samples. The temperature program was set from 30 to 500 °C with a heating rate of 10 °C/min under N_2_ flow. 

#### 2.4.5. Differential Scanning Calorimetry (DSC)

For Differential Scanning Calorimetry, a Perkin Elmer DSC-8000, Waltham, MA, USA, instrument was used to examine the thermal behavior of products. The samples were sealed in aluminum pans and placed in DSC. The analysis was conducted under nitrogen flow (20 mL/min) within a temperature range of 30–300 °C at a heating rate of 10 °C/min.

#### 2.4.6. Microscopic Observations of Formulations

The scanning electron microscopic (SEM) images of the cross-section of the lyophilized formulations were captured using a Hitachi TM-1000 Table-Top scanning electron microscope, Tokyo, Japan. Magnification is given in the figures. 

#### 2.4.7. Texture Profile Analysis (TPA)

TA.XT*plusC* Texture Analyser, Stable Micro Systems Ltd., Guilford, UK was used to determine the textural properties of solutions and gels. Gel formulations were transferred into 10 mL glass vials assuring smooth upper surfaces. The analytical probe of the device was compressed into each formulation two times to 15 mm depth at a probe speed of 2 mm s^–1^ with a 20 s delay period. Three replicate analyses were performed at room temperature for each formulation; each time there were provided the same conditions. Gel parameters such as hardness, cohesiveness, and adhesiveness were determined from the resultant force–time plot.

#### 2.4.8. Analytical Method for Drug Determination

The amount of Voriconazole was analyzed by using a high-pressure liquid chromatography (HPLC) system, Agilent 1200 Series, Santa Clara, CA, USA. The system consisted of a UV detector and ACE® C18 column (length of 25 cm, an internal diameter of 4.6 mm, a particle size of 5 μm, and a pore size of 110 Å) (Aberdeen, Scotland). A degassed solution containing 65% water and 35% acetonitrile (*v*/*v* %) was used as the mobile phase. The flow rate was set at 1 mL/min and the UV detection was set at 255 nm. The stock solutions of Voriconazole were prepared by dissolving 0.1 g of pure drug in 100 mL of phosphate buffer saline (PBS, pH = 7.4). The peak area correlated linearly with Voriconazole concentration in the range of 0.1–50 μg/mL (r^2^ = 0.9952). The validation of the method was accomplished on the specificity, stability, accuracy, and precision of the solution. The experiments were carried out in triplicate. The compounds were identified by comparing the retention times of the unknown peaks with the peaks of the reference standards. The retention time considered for Voriconazole was found at 19.6 min. Weighed amounts of lyophilized hydrogels were placed in a vial and 10 mL of phosphate buffer pH = 7.4 was added. The systems were stirred overnight to ensure a good extraction of the drug from the polymeric matrix. Volumes of 1 mL were sampled for the HPLC characterization. The loading percent was calculated taking into account the initial amount of Voriconazole loaded at the beginning of the experiment (1% against polymeric matrix). 

#### 2.4.9. In Vitro Release Study of Formulations

The dialysis bag diffusion technique was used to study the in vitro drug release of Voriconazole from prepared gels. Samples in gel state were weighted, then they were placed in the dialysis bag (cellulose membrane, molecular weight of 12,000–14,000 Da), hermetically sealed and immersed in 100 mL of PBS (pH = 7.4). The entire system (WiseBath®, Fuzzy Control System, Germany) was kept at 37 ± 0.5 °C with continuous magnetic stirring at 300 rpm. Samples were withdrawn from the receptor compartment at predetermined time intervals and the entire receptor phase was replaced with fresh medium at 37 ± 0.5 °C to obtain sink conditions. The amount of drug that was released was then determined by the HPLC method. The experiments were carried out in triplicate.

The drug release kinetics was evaluated with a semi-empirical Equation (2) based on Korsmeyer–Peppas model, which is applied at the initial stages (approximately 60% fractional release) [25].

(2) $$\frac{W_{t}}{W_{eq}}=k\times t^{n},$$

where *W_t_*/*W_eq_* represents the fraction of the drug released, *W_t_* and *W_eq_* are the absolute cumulative amount of drug released at time *t* and at equilibrium, respectively (in this case the maximum amount released in the experimental conditions used, at the plateau of the release curves), *k* is a constant incorporating the characteristics of the macromolecular drug loaded system, and *n* is the diffusional exponent characteristic for the release mechanism. 

In the equation above, value of *n* ≤ 0.5 indicates a Fickian diffusion mechanism of the drug from the hydrogel network, while a value 0.5 < *n* < 1 indicates an anomalous or non-Fickian behavior. When *n* = 1, a case II transport mechanism is involved with zero-order kinetics, while *n* > 1 indicates a special case II transport mechanism [26].

#### 2.4.10. Stability Studies

Gel formulations were stored at +40 °C and 75% Relative Humidity (RH) stability cabinets (TK 252 Test Cabinet; Nüve, Ankara, Turkey) in plastic cream-like recipients for two months. Samples were analyzed in terms of visual aspect (color, consistency), pH, drug content, and rheological characteristics after 15 days, 1 and 2 months. 

#### 2.4.11. Cell Culture and Cytotoxicity Tests

Human kidney proximal tubular epithelial cell line (HK-2, American Type Culture Collection-ATCC) was cultured in a 1:1 mixture of Dulbecco’s modified Eagle’s medium (DMEM): Nutrient mixture-F12 (DMEM: F12), supplemented with 2 mM glutamine and 5% fetal bovine serum (FBS). Mouse embryonic fibroblast cell line (NIH-3T3, ATCC) was grown in 10% FBS-supplemented DMEM medium.

The cytotoxic potential of the active substance and formulations was tested by MTT (3-[4,5-dimethylthiazol-2-yl]-2,5-diphenyltetrazolium bromide) assay. MTT reagent was reduced to dark blue formazan crystal form when incubated with viable cells. For experiments, cells were counted under a phase-contrast microscope and plated in 12-well plates at a density of 2.5 × 10^5^ cells/well incubated at 37 °C in a humidified atmosphere containing 5% CO_2_ for 18 h for cell attachment. Then, the cells were treated for 48 h with a 1:1000 dilution of the tested formulation. Cells treated with the medium were used as a negative control. Voriconazole (100 µg/mL) treated cells were used as a positive control. At the end of the incubation, the medium was removed, and the cells were incubated with DMEM containing MTT solution (5 mg/mL) for another 4 h. DMEM was removed, and blue-formazan crystals were dissolved in dimethyl sulfoxide, then transferred to a 96-well plate. The absorbance of the formazan solution was measured in a multi-plate reader (Varioskan Flash, Thermo) at 540 nm. The ratio of the absorbance of treated samples to the control (taken as 100%) was expressed as the percentage of cell viability. Cell viability was expressed as the percentage of formazan absorbance. Results were the mean ± standard deviation (mean ± SD) from at least three different experiments in triplicate. Statistical analysis was conducted by ANOVA followed by Tukey’s test for comparisons between groups. The *p* value of 0.05 was taken to indicate statistical significance (*p* < 0.05).

## 3. Results and Discussions

### 3.1. Optimization Studies

Semisolid systems are the most usual formulations for the delivery of drugs through the skin [23]. Regarding the physiological properties, semisolid dosage form should be non-irritating and not alter the membrane/skin functioning; it should also be miscible with skin secretion. Furthermore, dosage forms should be easily applicable on the surface, to efficiently release the drug and to possess high aqueous washing ability. 

Several formulations based on modified and unmodified chitosan/PVA were prepared in order to find out which one was optimal for the topical application of Voriconazole. Table 1 summarizes the gel formulations prepared for this purpose. 

Samples prepared with unmodified chitosan with PVA by the freeze–thaw technique acted as reference samples to understand whether the modification of chitosan was efficient in terms of its physical–chemical properties. 

Gels were widely accepted as being semisolid dosage forms. The strength of the gel played a more important role than the viscosity. For this, texture profile analysis (TPA) was used to measure the mechanical resistance of the sample to the penetration of probe. Parameters such as hardness, adhesiveness, compressibility, and elasticity were determined by TPA analysis and the results are listed in Table 2. 

Formulations for topical applications should possess high adhesiveness, ease of application to the surface, low hardness, and compressibility in order to easily handle the formulation from the container and apply to the mucosal area [23,24].

As listed in Table 2, the mechanical parameters calculated from TPA analysis showed that formulations based on CS-*g*-PNIPAAm/PVA 75/25 had high values of adhesiveness and cohesiveness while hardness was low, which make them suitable for topical application. By comparison, formulations with simple chitosan and PVA (CS/PVA) seemed to be strong gels which made it difficult to prepare semisolid dosage forms them. Following TPA studies, only certain samples were able to be measured via rheology in terms of flow behavior. 

**Figure 1:**
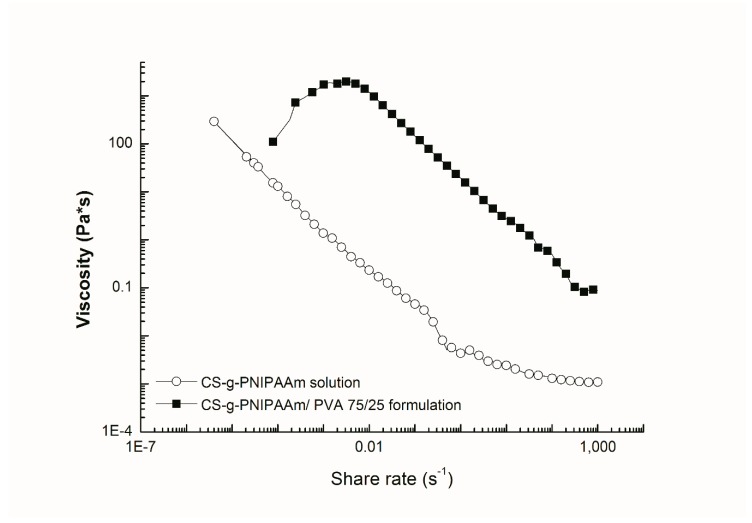
Flow behavior of the formulations prepared from grafted copolymer, with and without PVA.

In Figure 1, the flow behavior of two formulations is represented, one made only from the CS-*g*-PNIPAAm and the second one from cryogel prepared by freeze–thaw of CS-*g*-PNIPAAm/PVA 75/25.

Figure 1 shows that the simple solution of graft copolymer exhibited a clear shear thinning behavior at low share rates while the cryogel of the graft copolymer showed an initial shear thickening followed by a shear thinning behavior at higher share rates. The viscosity of the two formulations was obviously different, the crosslinked one was much higher. 

The outcome obtained from optimization studies was that the formulation based on graft copolymer CS-*g*-PNIPAAm/PVA with a composition of 75/25 *v*/*v* % should be further analyzed as a polymeric carrier of Voriconazole for topical application. 

### 3.2. Structure Identification of Hydrogels

#### 3.2.1. FTIR Spectra

**Figure 2:**
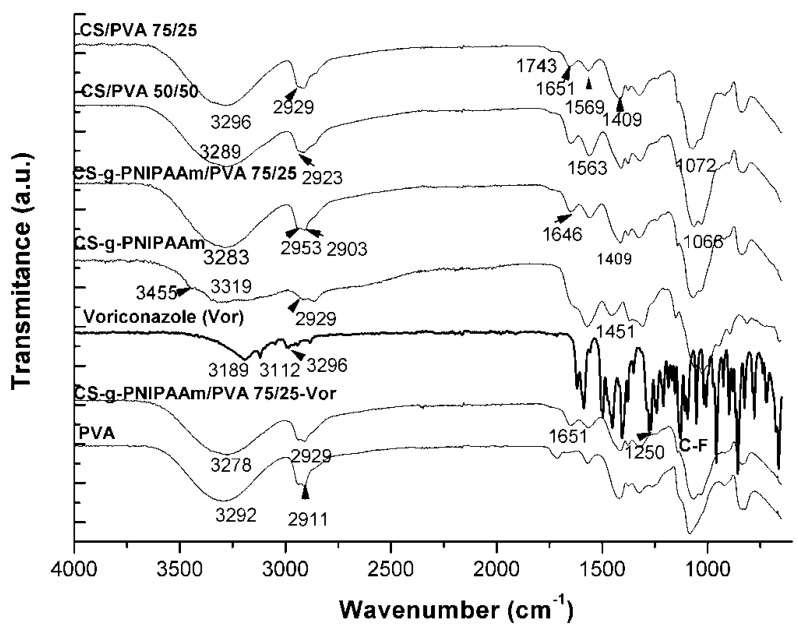
ATR–FTIR spectra of the freeze-dried hydrogels based on pure chitosan, PVA, and copolymer CS-*g*-PNIPAAm.

The FTIR spectra of PVA, chitosan, chitosan-graft-PNIPAAm and hydrogels are presented in Figure 2.

From the spectra, the characteristics peaks of chitosan, PNIPAAm, and PVA could be observed for the cryogels. The characteristic peaks of chitosan were found at 2921–2887 cm^−1^ (–CH_3_, –CH_2_), 1636 cm^−1^ (C=O stretch vibration), 1548 cm^−1^ (secondary amide), 1655 cm^−1^ (amide band I), 1068–1020 cm^−1^ (C–O stretching of saccharide moiety). The peaks of PNIPAAm moieties could be observed at 1454 cm^−1^ (–CH_3_) and 1641 cm^−1^ (O=C). Moreover, the absorption band around 3310 cm^−1^ became wider, indicating hydrogen bond formation between –OH and –NHCO groups. With the addition of PVA, the peaks observed at 3440 and 2921 cm^−1^ due to the –OH and CH_2_ stretching vibrations were found to be broader.

Voriconazole is a complex substance containing as active constituent (2R,3S)-2-(2,4-difluorophenyl)-3-(5-fluoro-4-pyrimidinyl)-1-(1H-1,2,4-triazol-1-yl)-2-butanol. Thus, Voriconazole’s absorption peaks in FTIR spectra were found in the region of 3200 cm^−1^ corresponding to the stretching vibrations of OH; peaks between 3000–2850, 1600–1400, and 1360–1250 cm^−1^ could be assigned to the alkane CH, C=C aromatic, and aryl C–N stretches, respectively. The characteristics vibration multiple bands for C–F were observed in the spectral region 1000–1200 cm^−1^. Due to the low amount of Voriconazole in the hydrogels (1 wt.%), its characteristic vibration bands could not be observed clearly. On the other hand, small increase could be observed in the intensity of overlapped regions between the hydrogels and drug. All spectra were interpreted by using available online accepted FTIR spectra databases [27].

#### 3.2.2. Thermal Behavior

**Figure 3:**
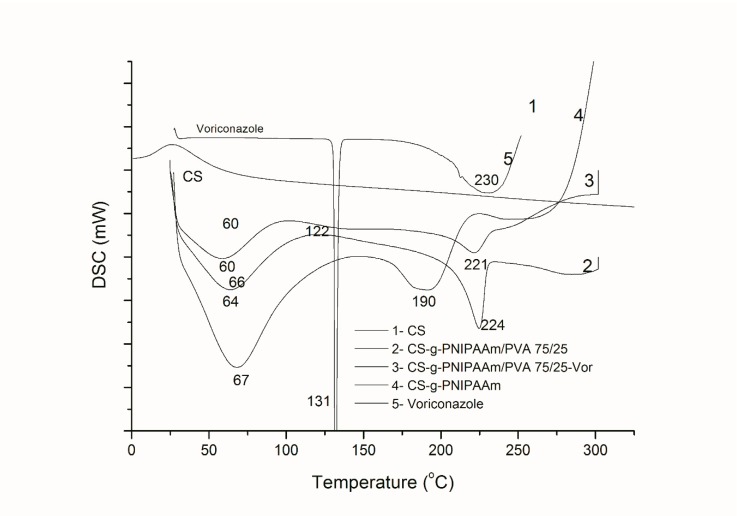
Differential scanning calorimetry (DSC) curves of the hydrogels of CS-*g*-PNIPAAm.

The thermal behavior of the selected gels in solid state was determined by means of DSC measurements and thermogravimetric studies. DSC curves are presented in Figure 3. 

As can be observed in Figure 3, the hydrogels prepared by the freeze–thaw technique presented a loss of free and bonded water at temperatures up to 100 °C. The melting peak of the graft copolymer was observed at 190 °C, whereas for hydrogels it was observed at around 221–224 °C The shifting of the melting peak to higher temperatures was due to the presence of PVA moieties since PVA as a crystalline polymer has a melting peak at 230 °C. The observation of one melting event in hydrogels also showed high interaction of PVA segments with graft copolymer verifying the efficiency of crosslinking.

**Figure 4:**
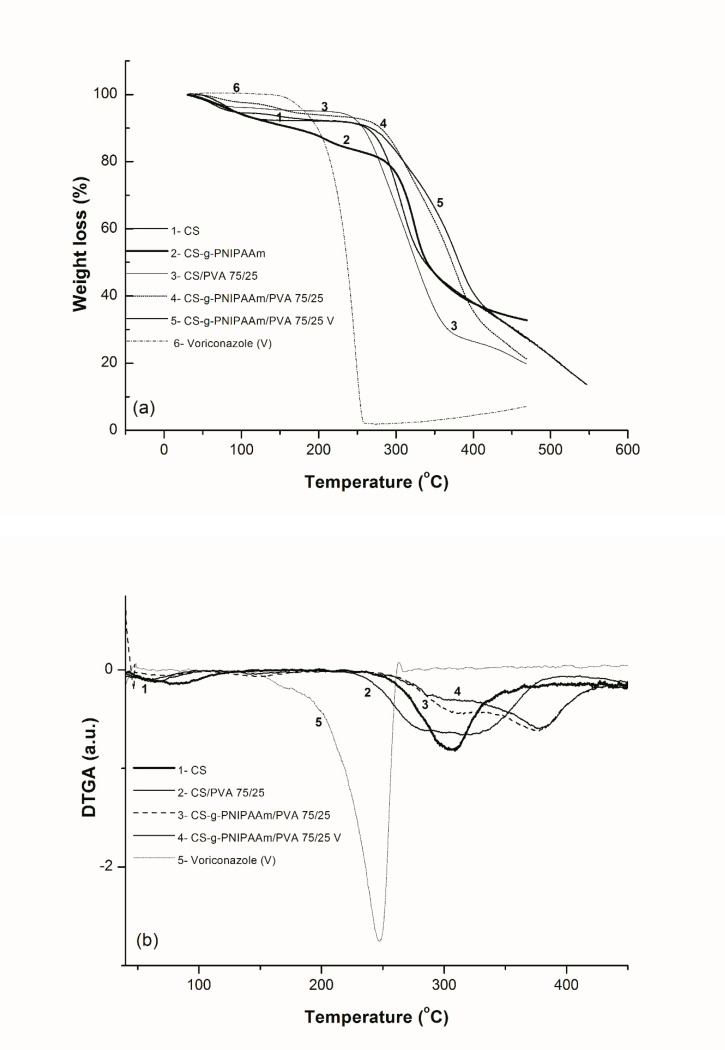
TGA/DTG curves of cryogels based on graft copolymer and pure constituents. (**a**) TG curves and (**b**) first derivative DTG curves.

Thermal degradation of hydrogels (Figure 4) showed two or three steps depending on the complexity of the structure. One main process was ascribed to the moisture loss up to 100 °C and the second thermal event was the main degradation step between 250 and 400 °C. The comparison of degradation peaks could be observed clearly in the first derivative of the TGA curves (DTG) (Figure 4b). The results showed that the hydrogels with PVA had higher thermal stability than simple graft copolymer and CS/PVA blend. This increase in thermal stability also verifies the strong interaction within the network of hydrogels.

#### 3.2.3. Swelling Ability of CS-*g*-PNIPAAm/PVA 

**Figure 5:**
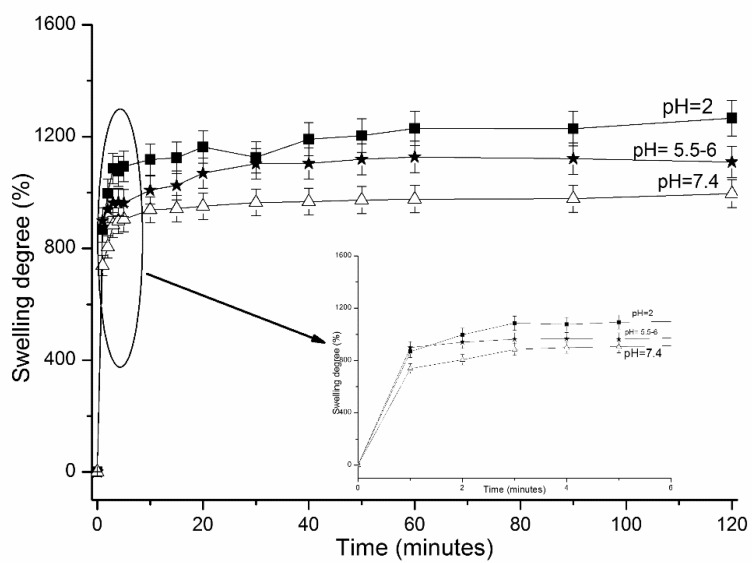
Swelling behavior of CS*-g-*PNIPAAm/PVA 75/25 hydrogel at different pH values.

The swelling profiles of selected CS-*g*-PNIPAAm/PVA hydrogels at different pH are presented in Figure 5.

A dependence on pH with respect to the swelling behavior was obtained. A maximum swelling degree (of 1266%) was determined at acidic pH due to the free –NH_2_ groups of chitosan which become protonated. With the increasing pH, simulating the physiological medium, a lower maximum swelling degree of 990% was found in phosphate buffer pH = 7.4. Similar pH-dependent swelling behavior of CS based hydrogels was also reported in a previous study [28]. 

#### 3.2.4. In Vitro Release of Voriconazole

Drug loading tests performed prior to in vitro release studies revealed that the matrices were able to uptake ~70 *w*/*w* % of the initial amount of drug (1% against polymeric matrix). 

**Figure 6:**
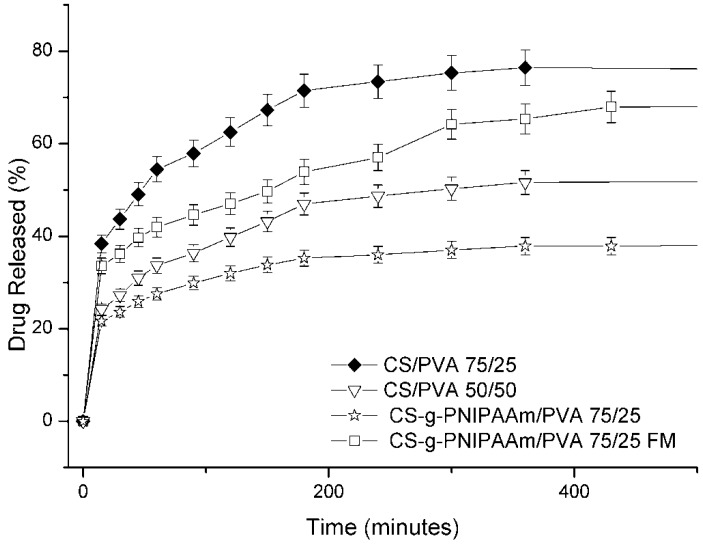
Voriconazole release profiles from different types of gel formulations.

In Figure 6, the in vitro release profiles of Voriconazole from the cryogels are presented.

For the hydrogel CS-g-PNIPAAm/PVA75/25 FM with Vor (Table 1), an initial burst effect was observed in the first 30 min, approximately 36% from the total amount released. The release profile reached a plateau after 5 h and maximum released amount was found to be 68%. On the other hand, the hydrogel CS-g-PNIPAAm/PVA 75/25 showed a much slower release profile. The released amount after 30 min was calculated as 23.6%. The release reached a plateau after 3 h and maximum released amount was 38% after 8 h of measurement time. It seems that preparation of the dosage form is important and influences the release profile possibly due to the better interaction of the drug with the matrix in the latter case. 

The matrices that were prepared as reference samples, particularly, CS/PVA 75/25 and CS/PVA 50/50 (Table 1), showed different profiles. Even though the drug was loaded during the cryogel preparation, the release of drug from the matrix was much faster. Moreover, with the increase of PVA amount within the hydrogel, the release rate and maximum released amount was lower. These outcomes supported the importance of the chitosan modification. The functionalized matrix had a more complex structure due to the primary and secondary amine groups on the side chains.

These results were also supported by the kinetic parameters calculated by the method of Korsmeyer–Peppas [25] (Equation (2)), and the results are summarized in Table 3. 

The *n* values lower than 0.5 indicate that the release mechanism of Voriconazole from cryogels follows a Fickian diffusion mechanism [26]. The small values recorded by the *n* parameter in the basic medium (pH = 7.4) are correlated with a slow drug release profile and are caused by the rate of polymer relaxation, which is much greater than the rate of drug diffusion. Both diffusion exponent, *n*, and kinetic constant, *k*, showed lower values at pH = 7.4. A decrease of the *k* values from 1.5 min^−0.18^ of CS/PVA 75/25 to 0.2 min^−0.17^ of CS*-g-*PNIPAAm/PVA 75/25 was associated with a slow drug release. The results obtained are comparable with those obtained in the case of a cryogel based on hyaluronic acid and PVA [19]. 

It can be concluded that CS-*g*-PNIPAAm/PVA 75/25 can be used for the type of systems to deliver low doses of drug, i.e., topical applications.

#### 3.2.5. Stability of CS*-g-*PNIPAAm/PVA Cryogels

**Figure 7:**
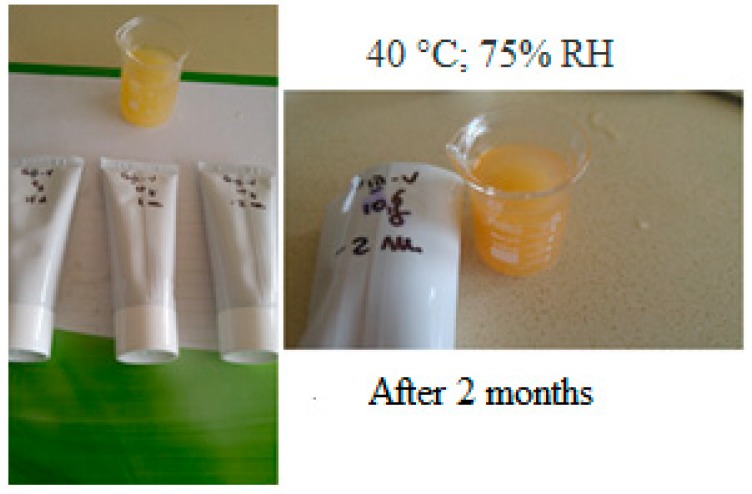
Image of formulation before (**left**) and after stability test (**right**).

The stability of the selected hydrogel loaded with Voriconazole, particularly CS-*g*-PNIPAAm PVA 75/25 was tested according to the storage guidelines. The stability tests were done under accelerated conditions for two months at 40 °C/75% RH ± 2 °C/±5% RH. The formulations after conditioning were characterized in terms of rheological and mechanical properties, pH, drug content, thermal stability, and morphological aspect. The results of the stability studies are presented in Table 4 and Figure 7.

After two months of aging, the compressibility capacity of the gel formulation was significantly changed. The other mechanical properties were slightly lower but still in the limit of acceptance as a semisolid dosage form. Moreover, the physical properties of the formulation were not changed except a slight change of the color to pink, probably due to the presence of chain transfer agent (CTA) moiety within the structure of the graft copolymer (Figure 7).

**Figure 8:**
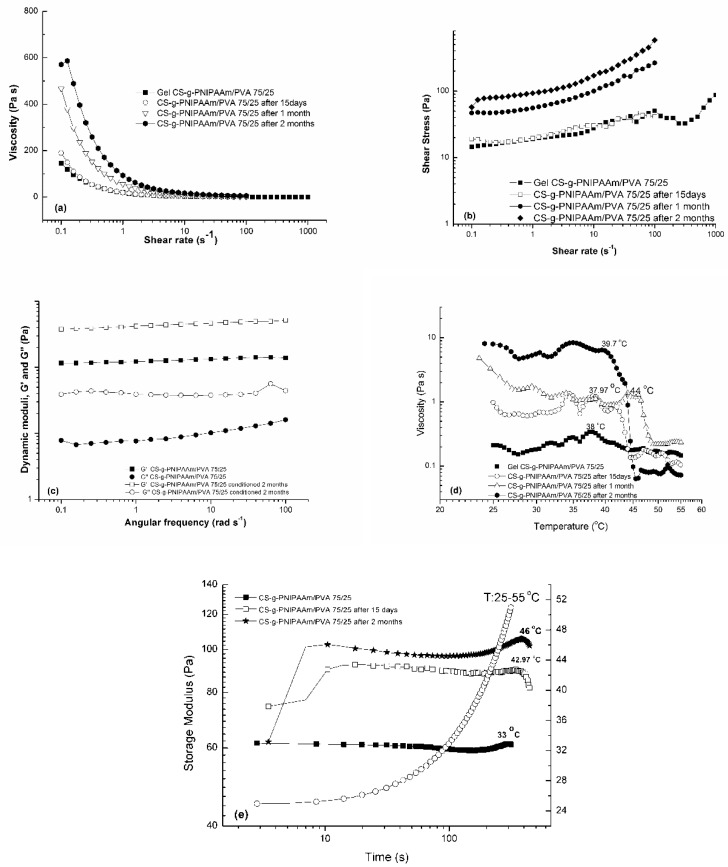
Rheological curves of CS-*g*-PNIPAAm/PVA 75/25. (**a**) Viscosity dependency on the share rate; (**b**) share stress–share rate curves; (**c**) variation of dynamic moduli, storage G’, and loss G’’ moduli with the angular frequency; (**d**) temperature sweep curves; and (**e**) time–temperature sweep tests.

Rheology was used to follow the change of viscoelastic character and pseudoelasticity of the gels. Figure 8a,b describes the flow behavior of the formulation before and after aging.

As shown in Figure 8a, the viscosity of the gel formulation at resting state showed an increase of the values for viscosity after each aging time. However, the flow behavior of the cryogels did not change and the same shear thinning behavior was observed. This can be due to the increase in interactions between the droplets at resting state leading to higher values of the flow resistance (viscosity). Usually polymers having high molecular weight have a tendency to entangle with their neighboring macromolecules in their three-dimensional network at “rest” state (low shear rates). However, during the shear process, the molecules are usually oriented in the shear direction by entangling to a certain extent which lowers their flow resistance [29]. With the increase of shear rate, a shear thinning behavior of the gels was very obvious, and, at higher share rates, a plateau exhibiting a Newtonian behavior was observed.

The pseudoplastic character was still visible even after applying load to the gels conditioned for two months (Figure 8b). Regarding the variation of dynamic moduli, storage, and loss moduli, in Figure 8c we noticed that there was an increase in the storage modulus of the aged hydrogel which could be correlated with the mechanical properties from TPA analysis (Table 4). This was probably due to the effect of entangling of the macromolecular chain as result of exposure to a temperature higher than the lower critical solution temperature (LCST) of PNIPAAm. 

The LCST of the gel was also determined by means of rheology (Figure 8d,e) via temperature and time sweep tests. From both tests, LCST and gelation temperatures were obtained in the range of 38–44 °C. It was observed that on exposing the formulations to aging, LCST and gelation temperatures shifted to higher values probably due to the entanglement of the chains. Gelation time was found to be approximately 2 min. 

#### 3.2.6. Microscopic Observations

**Figure 9:**
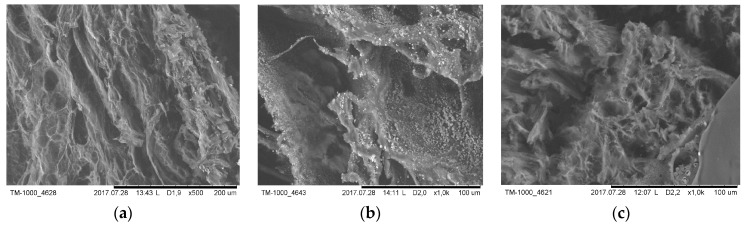
SEM images of freeze-dried CS-*g*-PNIPAAm/PVA 75/25. (**a**) Image of gel formulation before stability test, (**b**) image of gel formulation loaded with Voriconazole, (**c**) image of freeze-dried gel formulation after two months conditioning.

The sponge-like porous structures of freeze-dried cryogels was observed by means of SEM microscopy and the images are represented in Figure 9. 

Before aging, the gel showed a very clear porous structure after freeze-drying (Figure 9a). The presence of Voriconazole was also visible, shown as white dots that homogenously distributed throughout the matrix (Figure 9b). 

The aged cryogels after two months still had a porous structure; however, the pores were found to be smaller but still visible. 

#### 3.2.7. Cytotoxicity and Cell Culture Studies

Examination of the cytotoxicity is a crucial step for the development of novel formulations. For this purpose, CS-*g*-PNIPAAm/PVA 75/25 formulation was tested on two healthy distinct cell lines: In human kidney proximal tubular epithelial cell line (HK-2) and mouse embryonic fibroblast cell line (NIH-3T3). Epithelial and fibroblast cell lines are frequently used in such preliminary cytotoxic studies [30,31,32,33,34,35,36].

**Figure 10:**
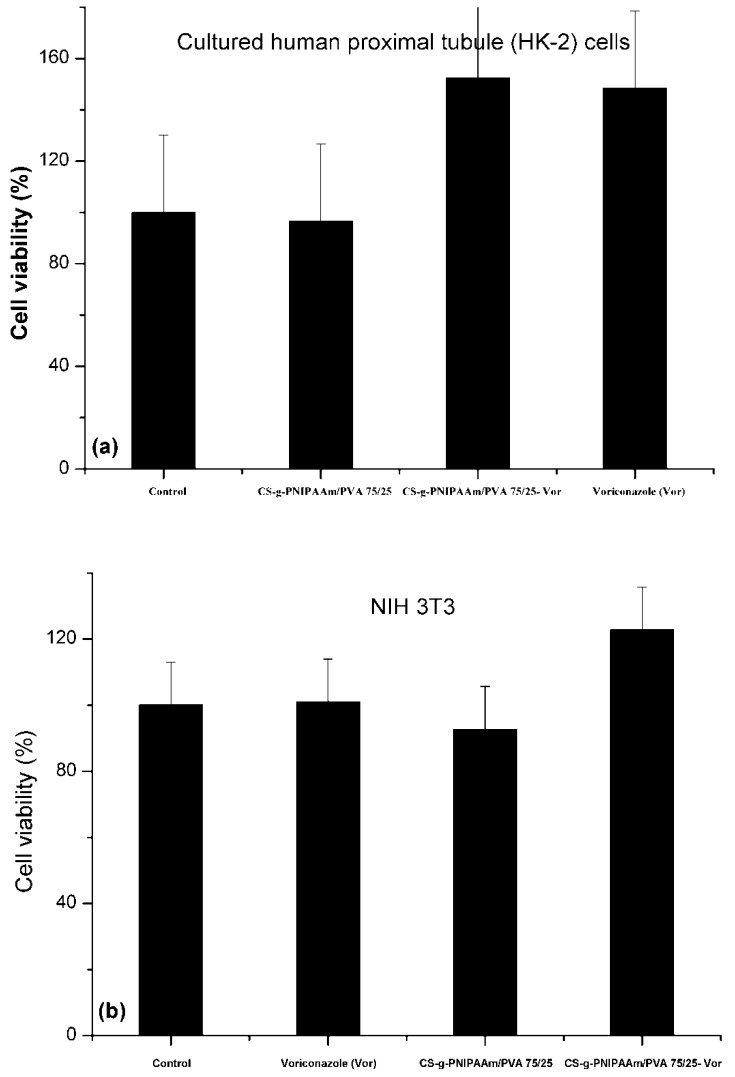
(**a**) Evaluation chart of cytotoxicity potential of the CS-*g*-PNIPAAm/PVA 75/25 based formulations prepared by MTT assay on human kidney tubular epithelial cells (HK-2). (**b**) Evaluation of cytotoxicity potential of CS-*g*-PNIPAAm/PVA 75/25 based formulations prepared by MTT assay on rat embryonic fibroblast cells (3T3). The bars show the value of “average ± standard deviation”. The values over the bars represent % cell viability in comparison to the control samples (* means the cell viability was increased in a statistical manner; *p* < 0.05; n = 4).

Cells were treated with CS-*g*-PNIPAAm/PVA 75/25 and Voriconazole-loaded formulation for 48 h. Furthermore, Voriconazole (100 µg/mL) treated cells were used as a positive control. At the end of the incubation time, the cell viability was measured by MTT assay (Figure 10a,b).

Figure 10a shows the cell viability rate of HK-2 cells. The results showed that unloaded CS-*g*-PNIPAAm/PVA 75/25 treatment did not affect the HK-2 cell viability compared to untreated control cells (96.59% ± 1.38%) (*p* > 0.05). However, Voriconazole and Voriconazole-loaded formulation led to a significant increase in HK-2 cell proliferation (148.39% ± 3.24%, 152.23% ± 4.01%, respectively) (*p* < 0.05).

The cell viability rate of NIH/3T3 cells is presented in Figure 10b. It was determined that unloaded CS-*g*-PNIPAAm/PVA 75/25 caused a slight decrease in NIH/3T3 cell viability (92.73% ± 2.24%) (*p* < 0.05). Voriconazole treated NIH/3T3 cells showed similar viability rate compared to control cells (100% ± 1.12%) (*p* > 0.05). On the other hand, Voriconazole-loaded formulation significantly induced cell proliferation rate in these cell line (122.79% ± 3.84%) (*p* > 0.05). 

Consequently, MTT assay results indicated that neither Voriconazole nor formulations loaded with Voriconazole had any cytotoxic effect at their tested concentrations on the established cell lines. Moreover, Voriconazole-loaded formulations showed similar cell viability to free Voriconazole treatment, which confirmed that the new formulation might be a promising carrier for Voriconazole.

## 4. Conclusions

A previously synthesized novel CS-*g*-PNIPAAm copolymer was used to prepare cryogels with PVA for topical application of an antifungal. The obtained cryogel solutions showed responsivity to pH and temperature proving their functionality. In vitro release profile of the cryogels exhibited a retarded release of the drug for 8 h. 

Texture profile analysis and rheological studies helped to establish that the best composition for the polymeric matrix was CS-*g*-PNIPAAm/PVA 75/25 showing defined pseudoplastic character, low hardness, high adhesion, elasticity, and compressibility. 

Stability tests of gel formulations for two months (40 °C and 75% RH) revealed that no significant change in structural characteristics was observed. The MMT assays on human kidney tubular epithelial cells and rat embryonic fibroblast cells showed that the formulation with and without drug did not show any cytotoxicity at the used concentration. 

Chitosan-graft-poly(N-isopropylacrylamide)/PVA cryogel might be used as polymeric vehicle for semisolid dosage forms in topical applications. 

## Figures and Tables

**Table 1:** List of formulations to be optimized.

Code	Formulation
CS-*g*-PNIPAAm/PVA 75/25 FM with Vor	Hydrogel CS-*g*-PNIPAAm physically crosslinked with PVA with a composition 75/25 *v*/*v* % by three cycles of −20/25 °C freeze–thaw; loading of Voriconazole was done by solution mixing after the hydrogel preparation
CS-*g*-PNIPAAm/PVA 75/25 with Vor	Hydrogel CS-*g*-PNIPAAm physically crosslinked with PVA with a composition 75/25 *v*/*v* % loading of Voriconazole was done during the preparation of hydrogel
CS/PVA 75/25	Physically crosslinked hydrogel between simple chitosan and PVA with a composition 75/25 *v*/*v* % by freeze–thaw −20/25 °C (three cycles)
CS/PVA 75/25 with Vor	Physically crosslinked hydrogel between simple chitosan and PVA with a composition 75/25 *v*/*v* % by freeze–thaw −20/25 °C (three cycles); loading of Voriconazole was done during the hydrogel preparation
CS/PVA 50/50	Physically crosslinked hydrogel between simple chitosan and PVA with a composition 50/50 *v*/*v* % by freeze–thaw −20/25 °C (three cycles)
CS/PVA 50/50 with Vor	Physically crosslinked hydrogel between simple chitosan and PVA with a composition 50/50 *v*/*v* % by freeze–thaw −20/25 °C (three cycles); loading of Voriconazole was done during the hydrogel preparation

CS-*g*-PNIPAAm—chitosan-graft-poly(N-isopropyl acrylamide); PVA—polyvinyl alcohol; Vor—Voriconazole.

**Table 2:** Texture profile analysis (TPA) results for the gels obtained for the pre-formulation studies.

System	Hardness	Compressibility	Adhesiveness	Cohesivity	Elasticity
CS-*g*-PNIPAAm/PVA 75/25 FM with Vor	0.948 ± 0.07	1.357 ± 0.13	−0.643 ± 0.01	0.89	0.97
CS-*g*-PNIPAAm/PVA with Vor	0.62 ± 0.02	1.20 ± 0.01	−0.57 ± 0.00	1.01	0.97
CS/PVA 75/25	7.56 ± 1.04	15.80 ± 4.57	−5.62 ± 0.38	0.77	0.90
CS/PVA 75/25 with Vor	2.48 ± 0.12	3.63 ± 0.33	−4.14 ± 0.22	0.92	1.11
CS/PVA 50/50	8.42 ± 0.81	16.67 ± 2.65	−6.78 ± 1.09	0.73	0.78
CS/PVA 50/50 with Vor	15 ± 0.12	26.56 ± 20.32	−1.82 ± 0.67	0.67	0.88

**Table 3:** Kinetic parameters calculated for the release profiles of Voriconazole from CS*-g-*PNIPAAm/PVA and CS/PVA cryogels.

Formulation	Percent Released from Total Amount in 6 h (%)	K (min)^−n^	n	R^2^
CS/PVA 75/25	76	1.50	0.18	0.98
CS/PVA 50/50	52	0.80	0.20	0.99
CS*-g-*PNIPAAm/PVA 75/25 FM	68	1.12	0.17	0.98
CS*-g-*PNIPAAm/PVA 75/25	38	0.20	0.17	0.97

**Table 4:** Parameters determined after conditioning of the gel formulation.

System	pH	Organoleptic Properties	Drug Content (μg/mL)	Mechanical Properties from TPA Analysis				
				H (N)	C (Ns)	E	Ch	A
**CS*-g-*PNIPAAm/PVA 75/25**	**5.0**	**Gel, pale yellow**	6.55	10.02	36.93	0.97	0.96	4.48
CS*-g-*PNIPAAm/PVA 75/25, after 15 days	4.85	Gel, pale yellow turned to light pink, no change in odor, no phase separation	6.85	14.44	16.80	0.98	0.84	4.47
CS*-g-*PNIPAAm/PVA 75/25, after 1 month	4.8	Gel, light pink-yellowish, no change in odor, no phase separation	6.78	10.30	18.92	0.94	0.86	4.80
CS*-g-*PNIPAAm/PVA 75/25, after 2 months	4.75	Gel, light pink-yellowish, no change in odor, no phase separation	6.58	12.09	18.52	0.95	0.91	4.93

H—hardness; C—compressibility; E—elasticity; Ch—cohesiveness; A—adhesion.
